# New developments in the pathology of malignant lymphoma: a review of the literature published from October 2014–December 2014

**DOI:** 10.1007/s12308-015-0240-9

**Published:** 2015-03-07

**Authors:** J. Han van Krieken

**Affiliations:** Department of Pathology, Radboud University Medical Centre, P.O. Box 9101, 6500 HB Nijmegen, The Netherlands

## Introduction

In this era of precision medicine, targets for new drugs are to be found across the range of tumors. Tissue microarrays are often used for that purpose, and for instance, Fernandez et al. [1] stained 395 lymphomas for Bruton’s tyrosine kinase protein expression showing that many B cell lymphomas, some T cell lymphomas, 14/16 nodular lymphocyte predominant, and 6/27 classic Hodgkin lymphoma (cHL) were positive. However, expression of a protein does not fully correlate with therapy response, so such studies can only serve as an initial screen. A next step is to study the effect of a drug in cell lines, like the work of Choudhary et al. [2], who investigated the effect of a Bcl-2 inhibitor on B cell lymphoma cell lines and also the acquired resistance after treatment. They showed that the B cell lymphoma cells upon treatment with Bcl-2 inhibition increased their expression of MCL-1 and Bcl-x, which resulted in resistance that could be overcome by inhibition of these factors. The authors indicate that a combination of these drugs might be a good rationale for therapy. However, I would be concerned when treatments would be based on physiological rather than pathological changes in neoplastic cells. In other words, such studies in follicular lymphoma seem to be more important than in lymphomas without *BCL2* alterations, even though the expression of the protein might be the same. Of course, in all cases, the final result needs to come from clinical trials.

## Biology of lymphoma

### Hodgkin lymphoma

Neoplastic cells have many dysregulated transcription pathways. By looking at the accessible sites of the chromatin in specific lymphoma types, Kreher et al. [3] discovered that interferon regulator factor (IRF) in cHL was a candidate for being specifically important for lymphoma development. In cell lines, they found that IRF5 is highly expressed and crucial for survival; they suggest that this factor has therefore a key tumor promoting role. cHL is characterized by the interplay between neoplastic cells and the microenvironment, and the amount of macrophages is an indicator for prognosis. Tudor et al. [4] analyzed 106 specimens using immunohistochemistry and found that high numbers of CD83 positive dendritic cells and low numbers of CD163 positive macrophages indicate improved survival. Furthermore, they found by RT-PCR in many samples increased levels of cytokines, both pro- and anti-inflammatory. Based on their data and cell-experiments, they propose a model by which cHL-cells induce maturation of dendritic cells, and vice versa, that macrophages promote immune evasion of cHL-cells.

### B cell lymphomas

Bauman et al. [5] searched in mantle cell lymphoma (MCL) for genetic alterations and found a common small deletion in *FBXO25* and monoallelic loss, resulting in lack of apoptosis through the mitochondrial protein HAX-1. The authors therefore conclude that *FBO25* is a tumor suppressor in MCL and *HAX-1* a protooncogene.

Tumor heterogeneity is a hot topic in cancer research. Spence et al. [6] used an original approach to study this phenomenon in follicular lymphoma (FL). They reasoned that activation-induced deaminase (AID) not only results in physiological somatic hypermutation (SHM) in the immunoglobulin genes (Ig), but also in other genes, referred to as aberrant somatic hypermutation (aSHM). Using ultradeep sequencing, they found single nucleotide variants in 12/12 FL with a median of 136 SHM and 53 aSHM. The number of variants at the *BCL2* locus correlated with other non-Ig loci, but not with Ig heavy chain gene (*IgH*). They conclude that genome-wide aSHM can be deduced by looking at the *BCL2* locus only.

Because they previously showed that about 40 % of extranodal marginal zone lymphoma (ENMZL) of the salivary gland express high affinity stereotypic rheumatoid factor B cell receptors, Bende et al. [7] investigated whether this is also the case in salivary gland biopsies of patients with Sjogren’s syndrome; they found this to be the case in 2/4 of such biopsies, but most of the clones they found had other B cell receptors. One of these two patients developed a diffuse large B cell lymphoma (DLBL) from the clone that expressed the rheumatoid factor recognizing receptor indicating a selection advantage of these receptors predisposing for lymphoma development.

Not only autoimmune disease predisposes for lymphoma development, but also hepatitis C infection. Arcaini et al. [8] investigated the relation between *NOTCH*-pathway mutations and hepatitis C-related lymphomas and found *NOTCH2* mutations in 9/46 (20 %) hepatitis C virus-positive DLBL patients, and *NOTCH1* mutations in 2/46 (4 %). By contrast, only 1/64 HCV-negative patient had a *NOTCH1* or *NOTCH2* mutation. The 5-year overall survival was 27 % for patients with a NOTCH pathway mutated DLBL versus 62 % for those without. Notch 2 is a critical membrane receptor for B cell functions and also displays various biological roles in lymphoma pathogenesis. Zhang et al. [9] show that a truncate *NOTCH2* mutation activates both the Notch 2 and the NF-κB signals and promotes the proliferation of B cell lymphoma cell lines. This proliferation was completely inhibited by ammonium pyrrolidine dithiocarbamate (PDTC), an NF-κB inhibitor. Simultaneously, PDTC also reduced the expression level of Notch 2 so that this may become a new therapeutical approach.

*NOTCH* induces *MYC*, connecting two signaling programs that are involved in lymphomagenesis. Ortega et al. [10] show that this relationship is bidirectional and that *MYC*, via a miRNA intermediary, modulates *NOTCH*. MicroRNA-30a (miR-30a), a member of a family of miRNAs that are transcriptionally suppressed by *MYC*, directly binds to and inhibits *NOTCH1* and *NOTCH2* expression. The activity of the miR-30a—*NOTCH*—*MYC* loop was validated in primary DLBL and T-ALL samples.

Another prime interest in B cell lymphomas is the NF-κB pathway, for which there are potential inhibitors for treatment. This pathway is activated in different lymphoma types through activating mutations. A subtype of DLBL with such mutations is primary central nervous system (CNS) DLBL, which has an aggressive clinical behavior. It has been seen as an immune privileged site due to the blood-brain barrier, and the question is how B cell receptor (BCR) activation is taking place. Akhter et al. [11] have analyzed the expression of 19 genes of the Toll-like receptor (TLR)/BCR pathway and targets in 20 cases of CNS DLBL. Compared to systemic DLBL, there was higher expression of TLR9, CD79B, CARD11, LYN, and BLNK. The B cell receptor molecules like BLNK and CD79B were also associated with higher expression of MYD88-dependent TLRs (TLR4/5/9). TLR overexpression is closely related with up-regulation of genes associated with BCR pathway like *CD79B/BLNK* and *CARD11*, which play an important role in NF-kB pathway activation. These results provide an important insight into the possibility of TLR and/or B cell receptor signaling molecules as possible therapeutic targets in CNS DLBL.

According to Gebauer et al. [12], primary mediastinal large B cell lymphoma has no mutations in *CARD11*, *CD79B*, or *EZH2*, based on 25 cases, but nevertheless may benefit from NF-κB inhibition since this pathway is also activated through B cell receptor signaling. Using this argument, any B cell lymphoma may benefit. Actually, these studies indicate that there is a need for a good marker for NF-κB activation.

### T cell lymphoma

Increasingly new targets for therapy are being discovered in B cell lymphomas, but fewer in T cell lymphomas although these have generally a poorer prognosis. Martin-Sanchez et al. [13] investigated the potential therapeutic value in T cell lymphomas of the Proviral Integration site of Moloney murine leukemia virus (PIM) kinases, which are overexpressed in peripheral T cell lymphomas (PTCL) and important mediators of cell survival. Inhibition of individual PIM kinases in a panel of 8 PTCL cell lines did not affect survival, partially because of a compensatory mechanism among the three *PIM* genes. In contrast, pharmacological inhibition of all PIM kinases strongly induced apoptosis in all PTCL cell lines, without cell cycle arrest, in part through the induction of DNA damage. Therefore, pan-PIM inhibitor (pan-PIMi) synergized with cisplatin. Importantly, pharmacological inhibition of PIM reduced primary tumoral T cell viability without affecting normal T cells ex vivo. Since anaplastic large cell lymphoma (ALK+ ALCL) cell lines were the most sensitive to the pan-PIMi, they tested the simultaneous inhibition of ALK and PIM kinases and found a strong synergistic effect in ALK+ ALCL cell lines. These findings suggest that PIM kinase inhibition could be of therapeutic value in a subset of PTCL.

Anaplastic large cell lymphoma (ALCL) is one of the most common T cell non-Hodgkin lymphomas and has two main subtypes: an anaplastic lymphoma kinase (ALK)-positive subtype characterized by ALK gene rearrangements and an ALK-negative subtype that is poorly understood. Xing et al. [14] recently identified recurrent rearrangements of the *DUSP22* locus on 6p25.3 in both primary cutaneous and systemic ALK-negative ALCLs. Since CCR8 has skin-homing properties it has been suggested to play a role in limiting extracutaneous spread of primary cutaneous ALCLs although overexpression of CCR8 has also been reported in systemic ALK-negative ALCLs. Using quantitative real-time PCR in frozen tissue and RNA in situ hybridization (ISH) in paraffin tissue showed higher CCR8 expression in ALCLs with *DUSP22* rearrangements than in nonrearranged cases. CCR8 expression was not associated with cutaneous presentation, cutaneous biopsy site, or cutaneous involvement during the disease course. These findings suggest that CCR8 expression in ALCL is more closely related to the presence of *DUSP22* rearrangements than to cutaneous involvement and that the function of CCR8 may extend beyond its skin-homing properties in this disease.

Weilemann et al. [15] investigated ALCL as well as focusing on the interferon regulatory factor 4 (IRF4), known to be highly expressed in both ALK-positive and ALK-negative cases. They show that ALCLs of both subtypes are addicted to IRF4 signaling, as knockdown of IRF4 by RNA interference was toxic to ALCL cell lines in vitro and in ALCL xenograft mouse models in vivo. Gene expression profiling after IRF4 knockdown demonstrated a significant downregulation of a variety of known *MYC* target genes. Furthermore, our analyses revealed that *MYC* is a primary target of IRF4, identifying a novel regulatory mechanism of MYC expression and its target gene network in ALCL. *MYC*, itself, is essential for ALCL survival, as both knockdown of *MYC* and pharmacologic inhibition of MYC signaling were toxic to ALCL cell lines. Collectively, these results demonstrate that ALCLs are dependent on IRF4 and *MYC* signaling and that *MYC* may represent a promising target for future therapies.

## Epidemiology of lymphoma

Although lymphomas are rarely seen in hereditary cancer syndromes, familial clustering suggests that there are susceptibility loci that enhance the risk to develop a lymphoma. Since lymphoma types are separate entities, association studies need to focus on well-defined entities such as FL, for which already such loci (HLA-variants) have been described. Skibola et al. [16] in a huge team effort, combined data from many patients and controls: 4523 case subjects and 13,344 control subjects of European ancestry. Five non-HLA loci were associated with FL risk: 11q23.3 near *CXCR5*; 11q24.3 near *ETS1*; 3q28 in *LPP*; 18q21.33 near *BCL2*; and 8q24.21 near *PVT1*. These findings further expand the number of loci associated with FL and provide evidence that multiple common variants outside the HLA region make a significant contribution to FL risk. Nevertheless, the increased risk is not substantial and not sufficient to use these data in screening or risk assessment. Cerhan et al. [17] performed a meta-analysis of similar studies in DLBL, which is a much less defined entity, totaling 3857 cases and 7666 controls of European ancestry. Five independent SNPs in four loci achieved genome-wide significance at 6p25.3 (*EXOC2*), 6p21.33 (*HLA-B*), 2p23.3 (*NCOA1*), and two independent SNPs at 8q24.21 (*PVT1*). Although the authors indicate that these data provide substantial new evidence for genetic susceptibility to this B cell malignancy and point to pathways involved in immune recognition and immune function in the pathogenesis of DLBL, it is indeed problematic that this analysis uses DLBL as one group. It is therefore hard to see how these data bring us further in understanding lymphomagenesis.

More interesting is the work of Muwazi et al. [18] although it is published in a much less prestigious journal (see editorial in this issue). They analyzed the seasonal variation in incidence of Burkitt lymphoma (BL), a well-defined entity with clear environmental aspects. All cases diagnosed from 1969 to 2006, from all over Uganda, at the Makerere University’s Department of Pathology, were evaluated with respect to monthly and rainy versus dry season prevalence. Although monthly frequencies varied considerably over the period, these differences were not significant; likewise, there was no statistically significant difference in the total number of Burkitt’s during the rainy compared dry seasons. These results indicate that mosquitoes are probable not directly involved in the pathogenesis.

Reference data from many patients in rare diseases can only be gathered by collaborative efforts. Munch-Petersen et al. [19] collected data from six eye cancer centers, over a period of 30 years, on 100 patients with ocular adnexal (OA)-DLBL. The median age was 70 years and 54 were female. The following three groups of patients with lymphoma could be identified: primary OA-DLBL (57.0 %), OA-DLBL and concurrent systemic lymphoma (29.0 %), and ocular adnexal lymphoma relapse of previous systemic lymphoma (14.0 %). Of 57 patients with primary OA-DLBL, 53 (93.0 %) had Ann Arbor stage IE disease, and 4 (7.0 %) had Ann Arbor stage IIE disease. According to the TNM staging system, 43 of 57 (75.4 %) had T2 tumors. Among all patients, the most frequent treatments were external beam radiation therapy with or without surgery (31.0 %) and rituximab-cyclophosphamide, hydroxydaunorubicin, vincristine sulfate, prednisone (CHOP), or rituximab-CHOP-like chemotherapy with or without external beam radiation therapy (21.0 %). The 5-year overall survival among the entire cohort was 36.0 %. Relapse occurred in 43.9 % (25 of 57) of patients with primary OA-DLBL. Increasing T category of the TNM staging system was predictive of survival in primary OA-DLBL, whereas the Ann Arbor staging system was not.

## Defining entities

### B cell lymphomas

As described above, patients with Sjogren syndrome have an increased risk on lymphoma but also have expanded B cell clones in the inflammatory phase of the disease. Johnsen et al. [20] analyzed biopsies from 21 Sjogren syndrome patients with lymphoma. GC-like structures (a term I actually do not like: germinal centers are very well defined, and it seems that primary follicles are included by the authors) in 17/40 (43 %) of the patients: 4/12 (33 %) with and 13/28 (46 %) without lymphoma. Surprisingly, monoclonal B cell infiltration was present in 5/12 patients (42 %) with and 5/28 patients (18 %) without lymphoma, which is in sharp contrast with other studies, in which virtually all lymphomas show clonality.

Gebauer et al. [21] collected 30 NMZL en performed microRNA profiling on low grade cases and cases that transformed to DLBL. While microRNA signatures of low-grade and transformed NMZL did not differ significantly, several microRNAs were differentially expressed between transformed NMZL and de novo DLBL. These are potentially relevant findings, because separating de novo DLBL from transformed cases may be clinically relevant.

Low grade B cell lymphomas primarily arising in the spleen are often difficult to classify, partly because they are rare, partly because there are overlapping features of the entities. Piva et al. [22] found recurrent mutations of the Kruppel-like factor 2 (*KLF2*) gene in 19/96 cases of splenic marginal zone lymphoma (SMZL); mutations were also found in other lymphoma types, but generally in lower frequencies: 16 % (4/24) of hairy cell leukemia (HCL), 11 % (17/154) of DLBL, 9 % (5/56) of NMZL, and 8 % (5/61) of extranodal marginal zone lymphomas (EMZL). They were rare or absent (frequency = 0–4 %) in the remaining lymphoma entities. Clipson et al. [23] investigated the same gene in the same number (coincidentally also 96!) of cases of SMZL but found more than twice as many mutations: 40, and rarely in other B cell lymphomas. In this study, *IGHV1-2* rearrangement and 7q deletion were primarily seen in SMZL with *KLF2* mutation, while *MYD88* and *TP53* mutations were nearly exclusively found in those without *KLF2* mutation. *NOTCH2*, *TRAF3*, *TNFAIP3,* and *CARD11* mutations were observed in SMZL both with and without *KLF2* mutation. Both studies indicate that *KLF2* mutation is the most common genetic change in SMZL, but sensitivity and specificity and the relation to other genetic alterations remains to be studied further.

Primary site of presentation is determining an entity for some specific lymphomas, like the skin, the mediastinum, or the testis. Primary presentation in the ovary is rare, and it is not clear that it represents a real subgroup. Sun et al. [24] studied 14 cases of which 12 were DLBL and 2 BL. The DLBL were all of the germinal center type and one had a *MYC* and *BCL2* break. The treatments and outcomes were highly variable, and it seems there are few arguments to categorize these as a separate entity.

Satou et al. [25] compared 33 EBV-positive BL with 117 EBV-negative cases from Japan. EBV-positive cases presented in older patients, more often in the tonsil and lymph nodes, whereas EBV-negative cases were more often located in the digestive tract. Based on these findings, the authors suggest that EBV-positive BL is an age-related disease. I am not sure that this concept really will be as successful as the EBV-positive DLBL of the elderly.

### T cell neoplasia

The diagnosis of T-lymphoblastic lymphoma/leukemia (T-LBL) can be a pitfall when it presents in the thymic regions: like thymocytes, the cells express TdT and Mib1 is almost 100 %. Of course, clonality testing can be helpful but takes time and is not everywhere available. Since the NOTCH pathway is frequently activated in T-LBL, Jegalian et al. [26] investigated whether an antibody to the NOTCH1 intracellular domain (N1ICD), which recognizes activated NOTCH1, might be a helpful diagnostic tool. Hyperplastic tonsil showed positivity in few scattered interfollicular lymphoid cells, suprabasilar epithelial cells, and endothelial cells. Thymocytes from non-neoplastic thymus were largely negative for N1ICD. All thymomas tested (*n* = 23) were negative for N1ICD, although epithelial cells and a small minority of thymocytes may be positive, requiring careful interpretation. All T-LBL cases (*n* = 16) were scored positive for N1ICD: 8 (50 %) of these showed diffuse and mostly strong immunoreactivity, whereas the remaining 8 (50 %) had less extensive positivity, but with consistently >25 % nuclear staining. Thus, N1ICD immunohistochemistry appears to be a useful method in distinguishing T-LBL from thymoma.

Also in T cell lymphomas, microRNAs were studied. Laginestra et al. [27] generated a microRNA profile of 23 peripheral T cell lymphomas (PTCL). The microRNA signature was compared with the gene expression profile of the same neoplasms. Two sets of microRNAs were identified that distinguished PTCL from angioimmunoblastic T cell lymphoma (AITL) and anaplastic large-cell lymphomas (ALCL) ALK-negative, respectively. This however seems to me a complicated way to reach a diagnosis.

Ng et al. [28] performed gene expression profiling in EBV-associated T/natural killer (NK)-cell lymphoproliferative disorder in children and young adults (TNKLPDC) in order to understand the molecular pathways deregulated in this disease and compared the results with those obtained in nasal-type NK/T cell lymphoma (NKTL). The molecular and phenotypic signature of TNKLPDC is similar to NKTL, with overexpression of p53, survivin, and EZH2. Down-regulation of *EZH2* in TNKLPDC cell lines led to an increase in apoptosis and decrease in tumor viability, suggesting that *EZH2* may be important for the survival of TNKLPDC cells and hence potentially a useful therapeutic target. Gene expression profiling revealed a distinctive enrichment of stem cell-related genes in TNKLPDC compared to NKTL. This was validated by a significantly higher expression of aldehyde dehydrogenase 1 (ALDH1) in TNKLPDC cell lines compared to NKTL cell lines.

Nakamoto-Matsubara et al. [29] looked for a better diagnostic tool to identify angioimmunoblastic T cell lymphoma (AITL) and separate it from PTCL using whole-exome and subsequent targeted sequencing. They identified G17V *RHOA* mutations in 60–70 % of AITL and AITL-like PTCL-NOS cases but not in other hematologic cancers, including other T cell malignancies. If confirmed, this could serve as a novel marker for AITL and AITL-like PTCL. It obviously raises the questions whether the latter are really not AITL.

### Cutaneous lymphomas

The spectrum of cutaneous CD30-positive lymphoproliferative disorders (LPDs) includes lymphomatoid papulosis and primary cutaneous anaplastic large cell lymphoma. Using whole-transcriptome sequencing, Velusamy et al. [30] identified a chimeric fusion involving *NPM1* (5q35) and *TYK2* (19p13) that encodes a NPM1-TYK2 protein containing the oligomerization domain of *NPM1* and an intact catalytic domain in *TYK2*. Fluorescence in situ hybridization revealed *NPM1-TYK2* fusions in 2 of 47 (4 %) primary cases of CD30-positive LPDs and was absent in other mature T cell neoplasms (*n* = 151). This is the first report of recurrent translocations involving *TYK2*.

Another difficult diagnosis in skin biopsies is early mycosis fungoides (MF). Ralfkiaer at al. [31] used a global quantitative real-time polymerase chain reaction platform to study microRNA expression in patients with early MF (*n* = 13), more advanced cutaneous (C)TCL (*n* = 42), and atopic dermatitis (AD, *n* = 20). Thirty-eight microRNAs were differentially expressed (≥twofold) in early MF vs. AD and 36 in early MF vs. more advanced disease. In early MF, additional miRs were significantly up-regulated compared to more advanced CTCL. In the 16 patients for whom detailed follow-up data were available, 72 miRs were found differentially expressed between patients with progressive vs. those with non-progressive disease. These data suggest that miR profiling in CTCL may be a key to improving both diagnosis and risk prediction, but this method that is not commonly available would need extensive validation.

Oschlies et al. [32] who have a rich pediatric lymphoma case collection describe their pediatric subcutaneous panniculitis like T cell lymphoma (SPLTCL), which is defined as α/β T cell receptor positive T cell lymphoma of CD8 positive cytotoxic T cells involving exclusively the subcutaneous tissue. SPLTCL is rare but has been shown to affect all age groups including children. The clinical course of this lymphoma depends on the presence or absence of a hemophagocytic syndrome (HPS), which has been reported to develop secondarily in approximately 20 % of SPLTCLs usually associated with an unfavorable outcome.

## Pitfalls in lymphoma diagnosis

Kaneko et al. [33] attempted to clarify the clinicopathological and immunohistochemical findings and presence or absence of Epstein-Barr virus (EBV) in tonsillar atypical interfollicular hyperplasia (AIFH). A total of 597 consecutive specimens from tonsillectomies performed between 1999 and July 2013 were reexamined. AIFH was identified in the tonsils in 12 (2.0 %) cases. These included seven males and five females, aged 3 to 19 years. Histologically, there was an expansion of the interfollicular areas by polymorphous infiltration resulting in distortion, but not obliteration of the normal tonsillar architecture. In some areas, the lymphoid follicles had hyperplastic germinal centers with ill-defined borders surrounded by sheet-like proliferation of polymorphous infiltrate showing a marginal zone distribution pattern. The infiltrate was composed of small to medium-sized (transformed) lymphocytes and immunoblasts accompanied by numerous plasma cells and plasmacytoid cells and resembling monocytoid B cells. The numerous immunoblasts were MUM1(+), CD10(−), BCL-6(−). EBER was positive in 9 of the 12 lesions. Although EBV-infection in tonsils is indeed a real pitfall, I do not think it is helpful to coin the term AIFH, which to me suggest a problem (atypical is often used in the setting of cancer) that is not real. Recognizing that transformed cells and distortion of the architecture can occur in reactive EBV lesions is more important that suggesting a new name for the lesion.

## Prognostic factors in lymphoma

Barreca et al. [34] used a novel single molecule RNA fluorescence based in situ hybridization to quantify *BCL2* RNA expression in FL cells. They discovered a highly variable expression in cells of a single lymphoma, and there was a correlation with the level of Ig RNA expression. The results are somewhat surprising since the protein levels are not very variable between the cells within a lymphoma. Having no experience with this specific technique, but with other RNA-based technologies, I wonder whether technical issues might be an issue. I am not sure that this is going to be a potential prognostic marker. There is more potential in the discovery of Correia et al. [35] who found that in FL, there is not only a translocation involving *BCL2* but there may also be mutations in the *BCL2* gene. The prevalence of *BCL2* coding sequence mutations was 12 % in FL at diagnosis and 53 % at transformation. The presence of these *BCL2* mutations at diagnosis correlated with increased risk of transformation and largely increased risk of death due to lymphoma.

The recognition of the importance of the immune response in cancer development has led to many articles on the prognostic impact of inflammatory cells in a variety of tumors. Nygren et al. [36] used flow cytometry in diagnostic MCL (*n* = 153) biopsies. MCL cases with diffuse and nodular histologic subtypes showed lower levels of T cells, especially CD4 T cells, than those with mantle zone growth pattern. Both CD3 and CD4 cells were lower in the nodular subtype than in mantle zone and in the diffuse compared with the nodular subtype. The CD4:CD8 ratios were inversely correlated to tumor cell proliferation. Higher levels of CD3 and CD4 T cells and higher CD4:CD8 ratios were associated with indolent disease. In multivariate analysis, the CD4:CD8 ratio correlated with overall survival independently of MCL-International Prognostic Index and high p53 expression.

Vajpayee et al. [37] correlated immunohistochemical expression of mTOR with germinal center and nongerminal center phenotype, *BCL2* and *MYC* expression, and International Prognostic Index (IPI) score in 31 patients with DLBL. Virtually, all patients with high mTOR scores had a germinal center phenotype, and within the germinal center subgroup, patients with high mTOR scores had higher IPI scores. Obviously, these data point to a potential indication of mTOR inhibitors for these patients.

Several studies have been performed using MYC immunohistochemistry to either define a lymphoma subgroup or as a prognostic indicator. Huang et al. [38] looked for *MYC*-expression in 69 ENMZL, including 42 cases without transformation, 20 cases with transformation and 7 cases of DLBCL with an ENMZL component. In total, 15/42 (35.7 %) cases were nuclear positive for *MYC* expression in the group without transformation, whereas 15/20 (75.0 %) cases were positive in the group with transformation and 4/7 (57.1 %) in the group of DLBCL + ENMZL. Therefore, *MYC* overexpression may play an important role in aggressive transformation, but the finding is surprising and needs confirmation.

Mahmoud et al. [39] took up the important challenge to validate *MYC* expression as a prognostic marker by looking at the reliability of its scoring by hematopathologists. Immunohistochemical evaluation of MYC protein expression in DLBL is a potential prognostic tool. Concordance of scoring in these studies was assessed among few pathologists from one institution by scoring tissue microarrays. In daily practice, MYC evaluation is performed on entire tumor sections by a diverse group of pathologists. Nine hematopathologists from two institutions scored whole-tissue sections of two sets of cases: a training set of 13 cases of DLBL and 4 cases of Burkitt lymphoma (BL) and a validation set of 18 cases of DLBL and 1 case of Burkitt lymphoma. MYC positivity was defined as ≥40 % of tumor cells demonstrating nuclear staining similar to prior studies. Discrepant cases from the training set were characterized by staining heterogeneity, extensive necrosis, or crush artifact and had mean scores within 15 percentage points of 40 %. Cases from the validation set that demonstrated any of these features were scored twice on two different days. Overall concordance was moderate (Kappa score, 0.68; *P* value < 0.001) with no significant change between the two sets (Kappa scores, 0.69 vs 0.67). Thirty-nine percent of cases were discrepant. The findings indicate that a significant number of diffuse large B cell lymphomas are inherently difficult to score due to staining heterogeneity. Careful scoring strategy in their study failed to improve concordance. This result is worrisome, but likely realistic: it shows that quantification by pathologists is inherently difficult and probably should be left to computerized approaches.

Coutinho et al. [40] took up the challenge to translate a gene expression profile into a prognostic tool based on immunohistochemistry. One has to realize that this is not yet very well possible for the identification of activated and germinal center types of DLBL. Diagnostic tissue from 309 patients was arrayed onto tissue microarrays. Results from 161 chemoimmunotherapy-treated patients were used for outcome prediction. By using computerized image analysis, they avoid the subjective interpretation by pathologists: positive cells, percentage stained area, and numbers of pixels/area were quantified. The concordance between two systems of image analysis was surprisingly high, supporting their applicability for immunohistochemistry studies. Patients with high density of CD3 positive cells and FoxP3 positive cells by both methods had a better outcome.

Chan et al. [41] profiled 148 genomes with 91 matching transcriptomes in a R-CHOP-treated DLBL cohort to uncover molecular subgroups linked to treatment failure. They found that deletions in RCOR1 were associated with unfavorable progression-free survival. They compiled a gene signature that identified a subgroup of patients with unfavorable overall survival. The prognostic significance of this 233-gene signature was reproduced in an independent cohort comprising 195 R-CHOP-treated patients. Additionally, within the IPI low risk group, the gene signature provided additional prognostic value that was independent of the cell-of-origin phenotype. This may therefore be a novel molecular subgroup of DLBL, although it remains unclear why simple detection of *RCOR1* deletions are not the way to go.

## Staging

It has been shown already many times that the amount of neoplastic cells in the bone marrow of FL patients indicate prognosis and that even low levels of these cells are prognostically relevant. Berget et al. [42] compared clonality testing, histology, and flow cytometry for detection of bone marrow involvement in FL patients. Indeed, patients with molecular but without morphological involvement had a poorer outcome. Surprisingly, patients with morphological and/or flow cytometrical involvement had the same outcome as patients without: this finding is so at odds with existing data that one must doubt the findings.

## Ancillary techniques

Challagundla et al. [43] measured CD200 expression levels in 505 peripheral blood, bone marrow, and lymphoid tissue biopsy specimens, including 364 cases positive for B cell leukemias and lymphomas. CD200 expression in chronic lymphocytic leukemia cases was as bright as or brighter than normal B cells in nearly all cases, while MCL cases were usually dim or negative. However, rare MCL cases (about 5 %) were moderately bright for CD200. MZL varied by subtype, with nodal cases brighter, splenic cases dimmer, and extranodal cases heterogeneous for CD200 expression. FL cells were brighter for CD200 in bone marrow specimens than in lymph nodes. DLBL of the non-germinal center type tended to be brighter for CD200 than those of the germinal center type, while BL were negative. They conclude that CD200 staining by flow cytometry can be useful in the differential diagnosis of B cell neoplasms and in their detection in the bone marrow, although the additional value to the standard approach was not given in this study.

Light chain restriction can be a helpful tool in defining B cell proliferations, but is not always easy to determine due to technical issues. Minca et al. [44] used ultrasensitive bright-field mRNA-ISH (BRISH) for in situ light-chain detection in cutaneous B cell lymphomas on 27 skin biopsies and excisions from patients with available results of B cell PCR-based clonality studies: 16 clonal B cell lesions and 11 non-clonal B cell proliferations. BRISH was successful in 15/16 clonal B cell lesions and 11/11 non-clonal proliferations. Light-chain restriction was detected in 15/15 clonal lesions and in 1/11 non-clonal proliferations (96.1 % overall concordance with clonality PCR). Therefore, ultrasensitive BRISH can successfully detect light-chain restriction in B-NHL from skin specimens and may be a useful adjunct ancillary tool.

Interpretation of the results of a clonality test is not always straightforward. Park et al. [45] used the Euroclonality/BIOMED2 primer set in 274 consecutive samples. Partly based on the guideline of Euroclonality (Langerak et al. [46]), they reported the results of 274 consecutive clonality cases and determined the interobserver reproducibility of individual primer set reactions and final results in a subset of 30 cases; 44/161 (27 %) B cell and 50/163 (31 %) T cell cases contained at least one abnormal peak. Of these, 29 (64 %) and 31 (62 %), respectively, showed clonal results in another primer set. Interobserver reproducibility was excellent for most primer sets and for final interpretations, but only fair to good for IGK V-J and TCRB D-J1 + 2 primer sets. A definitive diagnosis of lymphoma was rendered in 93, 20, and 6 % of B cell cases and 90, 42, and 14 % of T cell cases positive, indeterminate, or negative for a clonal population, respectively. The authors conclude that although abnormal (equivocal) peaks are frequently observed in routine practice, most of these contain clonal rearrangements in other primer sets, facilitating overall interpretation of final results with excellent interobserver reproducibility. This is in line with the experience that complementarity of the primer sets is a very important element in the Euroclonality approach. This is especially relevant when dealing with formalin-fixed, paraffin-embedded tissue. Moharrami et al. [47] also used the BIOMED-2 protocols to determine the clonality of *IGH* gene rearrangements in patients with lymphoma. PCR amplification was performed on FFPE of 50 patients with B cell lymphoma, which consisted of 11 cases with cHL, 25 cases of B-NHLs, and 14 cases of unclassifiable B-NHL. There was a clonal result in 96 % (24/25) of B-NHLs, whereas in cHL 82 % (9/11) of cases were clonal. These rates of clonality was not reached for a single target (FR1, FR2, FR3), which also confirms the importance of a complete approach.
